# Patient and health service factors associated with enrollment in a multidisciplinary pain rehabilitation program: a retrospective cohort study

**DOI:** 10.3389/fpain.2025.1455792

**Published:** 2025-04-10

**Authors:** Michael A. Bushey, Lindsay G. Flegge, Melissa Melendez, Elizabeth K. Harris, Flora M. Hammond

**Affiliations:** ^1^ Department of Psychiatry, Indiana University School of Medicine, Indianapolis, IN, United States; ^2^ Indiana University Health, Indianapolis, IN, United States; ^3^ Department of Physical Medicine & Rehabilitation, Indiana University School of Medicine, Indianapolis, IN, United States

**Keywords:** chronic pain, multidisciplinary pain clinics, health services research, pain rehabilitation, healthcare provider

## Abstract

**Introduction:**

Despite multidisciplinary pain rehabilitation programs (PRPs) being well-established as an effective treatment for chronic pain, the existence of such programs has been declining across the United States over recent decades.

**Objective:**

This study aims to identify factors associated with enrollment in a three-week, intensive outpatient PRP.

**Methods:**

This is a retrospective cohort study of all patient visits to a multidisciplinary pain evaluation clinic in 2023. The cohort was divided into those who did and did not subsequently enroll in a PRP program. Health service, demographic, and patient-reported outcome measures were compared between groups; continuous variables by independent samples Student's T-tests and categorical variables by chi-squared tests.

**Results:**

Of the 335 patients who had an evaluation in 2023, 48 went on to enroll in PRP (PRP-Yes group), and 287 did not (PRP-No group). Compared to PRP non-enrollers, the PRP-enrollers were more likely to have had a mental health (94% vs. 52%, *p* < .001) and physical therapy (94% vs. 48%, *p* < .001) assessment as part of their evaluation, had shorter lag times between their initial referral and medical evaluation [mean (SD) 43.5 (28.9) vs. 57.7 (41.7), *p* = .024], and had significantly greater anxiety, PTSD symptoms, somatic symptoms, and insomnia. Additionally, referral source, medical provider, and physical therapy provider seen differed significantly between PRP-enrollers and non-enrollers. PRP enrollment was not predicted by demographic variables including race, payer-type, or distance from the clinic.

**Discussion:**

Both personal and systemic factors were identified to be associated with enrollment in a three-week multidisciplinary PRP. These findings highlight variables worth considering for clinical and research programs looking to increase PRP enrollment.

## Introduction

Chronic pain is a highly prevalent condition, affecting 20% of adults in the United States [US; (1)]. Despite the existence of numerous treatment modalities, including medications, local injections, surgical repairs, restorative therapies, behavioral therapies, and complimentary approaches, chronic pain is highly persistent, with almost two-thirds who reported chronic pain in 2019 still reporting it a year later (2). Multidisciplinary pain rehabilitation programs (PRPs) first launched as a treatment option in the United States in the 1940s (3). The utility of this approach was immediately recognized, with expansion to an estimated 1,000 programs in the United States by 1990 (3). By integrating medical, psychological, and physical therapy approaches, PRPs are designed to address the complex nature of chronic pain (4). Research has consistently shown PRPs can significantly improve pain, functional ability, and quality of life (4–6). These programs have also been shown to decrease healthcare utilization (6, 7).

However, the availability of these programs began to decline in the 1990s, decreasing from 210 in 1998 to 84 in 2005 (8). The timing of this decline mirrors a rise in opioid prescriptions (9). In response to the combined opioid and pain crises, several US government agencies convened the Pain Management Best Practices Inter-Agency Task Force (10). Their published findings in 2019 emphasized the use of a multidisciplinary approach to treat chronic pain (10). In parallel, a resurgence of the multidisciplinary approach occurred within the US Veteran's Health Administration (VHA), with a ten-fold increased (from 2 to 20) in multidisciplinary pain treatment facilities from 2009 to 2019 (11). A report of outcomes on 931 patients to receive treatment through these programs found average effect sizes in pain outcomes ranging from medium to large (11). A qualitative interview study of 49 VHA providers identified several barriers to multidisciplinary care, such as competing pressures from expert guidelines, facility leadership, and patients (12). However, it is unclear if these insights are generalizable to other US health systems, necessitating research into barriers to multidisciplinary care in conventional health systems.

In addition to health system variables, patient-related barriers to accessing PRPs can significantly impact the utilization and effectiveness of these services. One prominent barrier is the stigma associated with chronic pain and mental health issues, which can discourage patients from seeking comprehensive treatment that includes psychological components (13, 14). Prior work has found that certain demographic characteristics, such as socioeconomic status and race, have led to inequitable access to pain treatment (15, 16). However, this prior work has not specifically focused on multidisciplinary pain programs *per se*, and there is a paucity of studies examining health service and other patient factors that specifically facilitate or impede enrollment in PRPs in conventional US health systems. The current study seeks to fill this gap to provide insights into this understudied area by comparing factors associated with successful enrollment of patients into a PRP among 335 patients evaluated in 2023. We hypothesized that we would be able to identify health services and patient variables that differed significantly between patients who did and did not subsequently enroll in a PRP.

## Methods

### Study design

This is a retrospective cohort study of all patients seen in the Indiana University Health (IUH) Pain Navigation Service (PNS) during the 2023 calendar year. Sample size for the study was determined by the clinic volume in the 2023 calendar year.

### Setting

The IUH PNS is a multidisciplinary pain evaluation clinic designed with the intent for patients to receive evaluations from a medical provider (nurse practitioner or physician with pain expertise), physical therapist, and mental health clinician. The clinic collects diagnostic information, pain history, and evaluates appropriateness for a variety of pain services. In particular, the PNS evaluation includes assessing the appropriateness for the IUH pain rehabilitation program (PRP), a multidisciplinary three-week intensive outpatient program. Typically, the medical, mental health, and physical therapy provider will confer to determine patient appropriateness for PRP. The PRP incorporates pain psychology, physical therapy, occupational therapy, yoga therapy, music therapy, massage therapy, dietetics, chaplaincy, social work, and peer support into full-day programming, 5 days per week. Appropriate referrals for the PNS are entered into a REDCap database (17, 18), where demographic and health service information is tracked. For this study, we analyzed data from all patients who completed a PNS medical appointment 1/1/2023–12/31/2023. Data capture was completed 6/1/2024, allowing a minimum of 150 days (for patients evaluated 12/31/2023) and up to 515 days (for patients evaluated 1/1/2023) for patients to have enrolled in PRP. Reviewing historical data of 117 patients enrolled in PRP since 2021, the mean lag time from PNS medical appointment to PRP start date was 103 days, with a range 11–727 days. At 150 days, 80% of historical patients would have been enrolled, and at 515 days, 98% would have been enrolled. This research was approved by the Indiana University Internal Review Board.

### Participants

Referrals to the PNS are screened by a trained nurse, who determines appropriateness of the referral. The primary criteria for appropriateness include that pain is chronic (i.e., has been present for at least 6 months) and that the referral is not specifically for ongoing chronic management of opioid therapy. To reduce the potential for bias, all patients who had a PNS medical visit were included in this analysis; there were no exclusion criteria.

### Data sources

The Pain Navigation Clinic maintains a REDCap clinical database in which certain information about appointments are tracked (see Variables below for more information). Before or during their Pain Navigation visit, patients are asked to fill out a health questionnaire through REDCap. All variables used for this study were collected from the REDCap database.

### Variables

Participants were grouped based upon the outcome of PRP enrollment (patients who attended at least one day of PRP have an enrollment date entered into REDCap). Based on this outcome, the sample was divided into two groups, “PRP-Yes” and “PRP-No”. All other variables were assessed as potential predictors of this outcome. Health service variables entered into the REDCap database include patient date of birth (DOB) and street address, primary insurance, referral date, referring department, PNS medical appointment initial scheduling day, PNS appointment date for medical, mental health, and physical therapy appointment, PNS provider seen, and PRP start date. Derived variables for this analysis included age at PNS medical appointment (PNS date – DOB), PNS lag (PNS medical appt date – referral date), distance from clinic (calculated by measuring the driving distance between patient and clinic zip code using Google Maps), and binary variables on whether patients attended any of the three PNS appointments and whether they enrolled in (i.e., went to at least one day of programming) PRP. This latter variable was used to divide the sample into two groups, the “PRP-Yes” group, and “PRP-No” group.

Patients were provided with baseline questionnaires at both their PNS medical and mental health appointments. Questionnaires could be filled out directly into REDCap from phone/tablet/computer, or on paper and transferred to REDCap by clinic staff. Measures that appeared on both questionnaires (described in more detail below) included: demographics, PEG (19), GAD-2/7 (20, 21), and PHQ-2/9 (22, 23). If a measure was completed at multiple appointments, the version from the medical appointment was used. Measures only administered at the medical visit included: Fibromyalgia Diagnostic Questionnaire (24), Opioid Use Disorder (OUD) symptom checklist (25), and Patient-Reported Outcomes Measurement Information System (PROMIS) Physical Function 12a (PROMIS-PF) (26). Measures administered only at the mental health visit included the Pain Catastrophizing Scale [PCS (27);], Insomnia Severity Index (ISI) (28), Primary Care PTSD Screen for DSM-5 (PC-PTSD-5) (29), and PROMIS Social Roles & Abilities (PROMIS-SF) scale (30).

For demographic variables, multiple choice questions assessed gender, birth sex, sexual preference, race, ethnicity, highest education attainment, marital status, primary employment category, income sufficiency, and pain duration. Four additional questions asked patients to estimate the approximate amount of time they spend each week working for pay, working around the home (e.g., laundry, childcare), at school or doing coursework, or volunteering. Response options for these four questions included: None, 1–10 h, 11–20 h, 21–40 h, and >40 h.

The PEG scale is a 3-item patient-reported outcome measure that evaluates pain severity (P) and pain interference with enjoyment of life (E) and general activity (G). Each item is scored 0–10, with total score being an average of the 3 items (0–10). Higher scores represent higher pain burden.

The Patient Health Questionnaire-9 (PHQ-9) is a 9-item measure consisting of questions that assess the severity of depressive symptoms over the past 2 weeks based on Diagnostic and Statistical Manual of Mental Disorders (DSM-IV) criteria. Each item is scored 0–3, with total scores ranging 0–27. Higher scores represent more severe depression. The PHQ-2 is a screening measure utilizing the first 2 questions of the PHQ-9. Our questionnaire utilizes branched logic whereby patients who score ≥2 on the PHQ-2 are provided the additional seven questions to answer.

The Generalized Anxiety Disorder-7 (GAD-7) is a 7-item measure that assesses the severity of anxiety symptoms in individuals over the past two weeks. Each item is scored 0–3, with total scores ranging 0–21. Higher scores represent more severe anxiety. The GAD-2 is a screening measure utilizing the first 2 questions of the GAD-7. Our questionnaire utilizes branched logic whereby patients who score ≥2 on the GAD-2 are provided the additional five questions to answer.

The Fibromyalgia Diagnostic Questionnaire consists of 3 parts: The Widespread Pain Index (WPI), Symptom Severity Score (SSS), and an item asking whether symptoms have been present ≥3 months. The WPI asks patients to score areas (from a list of 19 regions) where they have felt pain over the past week, with scores ranging 0–19. The SSS is a two-part assessment. Part A contains 3 multi-response items measuring fatigue, sleep effectiveness, and cognition. Scores on part A range 0–9. Part B asks patients to check symptoms they have experienced over the past week from a menu of 41 items. Scores on part B range 0 to 3 (0 = 0 symptoms, 1 = 1–10, 2 = 11–24, 3 = 25 or more symptoms). Part A + Part B = the total SSS score. Fibromyalgia diagnostic criteria are met if symptoms have been present for ≥ 3 months and [(WPI ≥ 7 and SSS ≥ 5) OR (WPI = 3–6 AND SSS ≥ 9)].

The Opioid Use Disorder (OUD) Symptoms Checklist is an 11-item measure asking patients to report on the eleven DSM-5 diagnostic criteria for OUD. A screening question prior to this measure asks whether patients have taken any opioids in the preceding year, and only those who answer yes are presented the 11-items. An additional item asks patients if they are currently prescribed opioids, as the items addressing tolerance and withdrawal are not counted toward the final score in such patients. Scores range from 0 to 11 (0–9 in patients prescribed opioids), with scores ≥2 representing a diagnosis of mild OUD, and higher scores representing greater OUD severity.

The Patient-Reported Outcomes Measurement Information System (PROMIS) Physical Function 12a (PROMIS-PF) measures physical function. An introductory question asking whether the patient can walk 25 feet on a level surface determines whether all 12 or only 6 items are asked. Each item is scored 1–5, with total scores ranging 6–60. Higher scores represent a higher level of physical function. Raw scores were used for this study.

The PROMIS Social Roles & Abilities scale is an 8-item measure that assesses an individual's perceived limitations and abilities in fulfilling social roles. Each item is scored 1–5, with total scores ranging 8–40. Higher scores represent a greater ability to participate in social roles and activities. Raw scores were used for this study.

The Insomnia Severity Index (ISI) is a 7-item self-report measure designed to assess the nature, severity, and impact of insomnia symptoms on an individual's daily functioning. Each item is scored 0–4, with total scores ranging 0–28. Higher scores represent more severe insomnia.

The Pain Catastrophizing Scale (PCS) is a 13-item self-report questionnaire that measures an individual's tendency to engage pain catastrophizing. Each item is scored 0–4, with total scores ranging 0–52. Higher scores represent more severe pain catastrophizing.

Primary Care PTSD Screen for DSM-5 (PC-PTSD-5) begins with a stem question asking if patients have ever experienced a traumatic event (with examples). Those answering “yes” are provided 5 additional yes/no questions asking about different symptoms of PTSD in the past month. Scores range 0–5, with higher scores reflecting more PTSD symptoms.

### Statistical methods

Descriptive analyses included frequencies, means, and standard deviations. Between group differences in continuous variables were compared via independent samples Student's T-tests, while chi-squared tests were used to compare between group categorical variables. Where possible, categorical groups containing five or fewer patients were combined with other groups before analysis. A *P*-value ≤.05 was considered statistically significant. Patients who enrolled in PRP were included in the “PRP-Yes” group, while patients who did not enroll in PRP were included in the “PRP-No” group. For symptom measures, any scales with unexpected missing values were excluded from analysis. “Expected” missingness occurred for scales that include branch logic such that not all items are presented to all patients. The predominant reason for missing values were technical errors (e.g., accidentally skipping a question) and clinic logistical issues (i.e., not having a systematic approach to ensuring measures are completed). These forces are independent from patient-specific factors and were considered missing-at-random. Analyses were conducted in statistical software developed by International Business Machines (IBM SSPS).

## Results

### Participants

A total of 335 participants attended a PNS medical appointment in 2023**.** Age in our sample ranged from 18 to 89 years, with mean (SD) of 47.5 (15.2) years. For the patients who completed questionnaires of demographic data (44%–50% of the sample), 75% were female, 83% straight, 76% were White and 15% were Black, with 96% of respondents being non-Hispanic. Most respondents (78%) reported pain duration of at least 5 years, with 53% reporting pain duration of more than 10 years. Most respondents (94%) had at least a high school degree, with an even distribution of terminal degrees between high school and graduate (15%–22% in each group). Most respondents (64%) did not identify as full- or part-time employed, with Social Security Disability Insurance (SSDI) being the largest represented employment category (21% of respondents), and 24% of respondents reported not making enough income to make ends meet. A more granular assessment of patient-reported activity shows that most patients estimate spending <10 h per week on all four key activities assessed (work for pay 62%, work at home 70%, schoolwork 90%, volunteering 95%), suggesting low levels of overall activity.

Participant home address distance from clinic ranged from 0 to 237 miles, with mean (SD) distance from clinic of 30.0 (37.1) miles. Of note, one patient had a home address listed in Alaska and was excluded from distance analysis as it was assumed this was an old address and inclusion of this outlier would have provided a skewed representation of travel distance. Distance from clinic was not significantly different (*P* = 0.83) between patients who did and did not attend PRP [31.0 (46.9) vs. 29.8 (35.3), respectively].

### Main results

**Figure 1:**
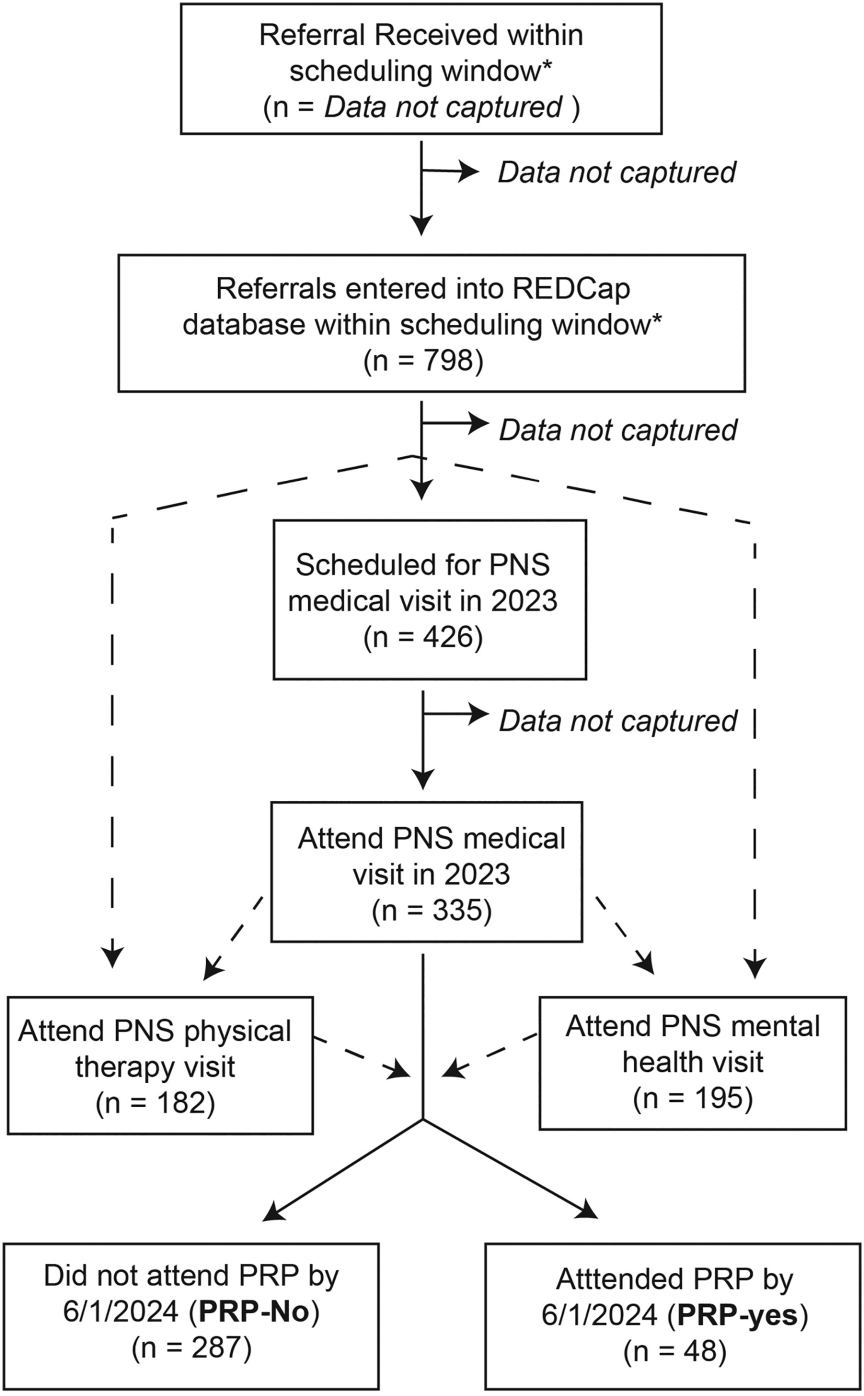
Participant flow diagram. Dashed lines represent optional flow pathways, since scheduling of physical therapy and mental health evaluations could be done in parallel or sequentially with scheduling the medical visit. *Based on an analysis of scheduling lag data (see Supplemental Figure), we defined “referrals within the scheduling window” as referrals received in 2023 OR referrals received after August 2022 but not scheduled for a visit in 2022.

Of the 335 patients who had a PNS medical visit in 2023, 48 went on to enroll in PRP (PRP-Yes group), and 287 did not (PRP-No group) (Figure 1). Health services information and demographic characteristics of our sample are shown in Table 1 (continuous variables) & Table 2 (categorical variables). In our overall sample, 195 (58%) had a mental health visit, 182 (54%) had a PT visit, and 139 (41%) had all three (medical, PT, and mental health) visits. Attending mental health, PT, or all three visits was highly associated with enrolling in PRP (*P* < .001 for each). Only 2% of patients who did not have both mental health and PT visits attended PRP, whereas 23% of patients with a mental health appointment, 25% of patients with a PT appointment, and 32% of patients who had all three visits attended PRP. The lag time between initial referral and PNS medical visit differed significantly between groups (*P* = .02), with PRP enrollers having a mean lag of 43.5 (28.9) days compared to a lag of 57.7 (41.7) days in non-attenders.

**Table 1:** Comparison of continuous variables between PRP non-enrollers (PRP-No) and enrollers (PRP-Yes).

Variable	Total		PRP-No		PRP-Yes		
	*N*	Mean (SD)	*N*	Mean (SD)	*N*	Mean (SD)	*P*-value
Age (years)	335	47.5 (15.2)	286	48.8 (24)	48	45.6 (16.7)	0.382
Distance (miles)	334	30 (37.1)	286	29.8 (35.3)	48	31 (46.9)	0.831
PNS med visit lag (days)	335	56.7 (36.5)	287	57.7 (41.7)	48	43.5 (28.9)	0.024
Pain (PEG)	212	7.0 (1.9)	176	7.0 (2.0)	36	7.2 (1.4)	0.561
Depression (PHQ)	221	9.5 (7.7)	182	9.2 (7.8)	39	11 (7.5)	0.172
Anxiety (GAD)	211	7.5 (6.3)	175	7 (6.3)	36	9.8 (6.4)	0.018
Catastrophizing (PCS)	134	24.1 (12.9)	101	23.8 (13.9)	33	25 (9.1)	0.641
Physical Function (PROMIS-PF)	158	20 (11)	135	20.2 (10.6)	23	19.3 (13.3)	0.730
Social Function (PROMIS-SF)	136	17 (7.4)	103	17.3 (7.6)	33	15.7 (6.4)	0.272
Widespread pain index (WPI)	335	3.3 (5.1)	287	3.1 (4.9)	48	4.5 (6.2)	0.090
WPI # of regions	335	1.4 (2)	287	1.4 (2)	48	1.7 (2.2)	0.301
Symptom Severity Score (SSS)	167	6.8 (2.6)	144	6.6 (2.5)	23	8 (2.4)	0.019
Insomnia (ISI)	124	14.8 (7.3)	95	14.1 (7.3)	29	17.2 (6.9)	0.045
PTSD (PC-PTSD-5)	132	2.2 (2)	101	2 (2)	31	2.9 (1.7)	0.015
OUD Sx Checklist	95	0.5 (0.9)	82	0.5 (0.9)	13	0.7 (1.1)	0.396

*P*-values were calculated using Student's T-test for independent samples.

PRP, pain rehabilitation program; SD, standard deviation; PNS, Pain Navigation Service; PEG, 3-item (Pain,Enjoyment,General activity) scale; PHQ, Patient Health Questionnaire depression measure; GAD, Generalized Anxiety Disorder measure; PM&R, physical medicine & rehabilitation; PCS, Pain Catastrophizing Scale; PROMIS, Patient-Reported Outcomes Measurement Information System; PF, physical function; SF, social function; PC-PTSD-5, Primary Care Post-Traumatic Stress Disorder Screen for DSM-5; OUD Sx, Opioid Use Disorder Symptom.

**Table 2:** Comparison of categorical variables between PRP non-enrollers (PRP-No) and PRP-enrollers (PRP-Yes).

Variable	Total	PRP-No	PRP-Yes	*P* -value
	*N*	*N* (%)	*N* (%)	
Payer				
Commercial	124	105 (37%)	19 (40%)	0.732
Medicaid	98	87 (30%)	11 (23%)	
Medicare	104	87 (30%)	17 (35%)	
Other	9	8 (3%)	1 (2%)	
Referring Dept				<0.001
Neurology	70	65 (23%)	5 (10%)	
Primary Care	62	56 (20%)	6 (13%)	
PM&R or PT	46	38 (13%)	8 (17%)	
Medical Genetics	42	38 (13%)	4 (8%)	
Pain Management	29	28 (10%)	1 (2%)	
Rheumatology	29	21 (7%)	8 (17%)	
Psychiatry	25	15 (5%)	10 (21%)	
Medical Miscellaneous	18	15 (5%)	3 (6%)	
Other	14	11 (4%)	3 (6%)	
PNS Medical provider				0.006
1	44	43 (15%)	1 (2%)	
2	173	139 (48%)	34 (71%)	
3	118	105 (37%)	13 (27%)	
Mental Health visit				<0.001
No	140	137 (48%)	3 (6%)	
Yes	195	150 (52%)	45 (94%)	
Mental Health provider				0.367
1	66	56 (35%)	10 (24%)	
2	91	71 (44%)	20 (48%)	
3	47	35 (22%)	12 (29%)	
PT visit				<0.001
No	153	149 (52%)	3 (6%)	
Yes	182	138 (48%)	45 (94%)	
PT provider				0.038
1	128	93 (67%)	35 (78%)	
2	27	24 (17%)	3 (7%)	
3	18	16 (12%)	2 (4%)	
Other	10	5 (4%)	5 (11%)	
All 3 visits				<0.001
no	196	193 (67%)	3 (6%)	
yes	139	94 (33%)	45 (94%)	
Race				0.689
Black	24	19 (14%)	5 (19%)	
White	122	103 (77%)	19 (73%)	
Other	13	11 (8%)	2 (8%)	
Ethnicity				0.599
Non-Hispanic	158	133 (98%)	25 (96%)	
Hispanic	4	3 (2%)	1 (4%)	
Gender				0.462
Female	127	108 (77%)	19 (68%)	
Male	34	27 (19%)	7 (25%)	
Other	8	6 (2%)	2 (4%)	
Sex				0.24
Female	125	106 (80%)	19 (70%)	
Other	34	24 (18%)	8 (30%)	
Sexual preference				0.656
Straight	134	112 (82%)	22 (85%)	
Other	28	24 (18%)	4 (15%)	
Education				0.256
<High School	10	9 (7%)	1 (4%)	
High School degree	27	22 (16%)	5 (19%)	
Some College	40	35 (26%)	5 (19%)	
Associate degree	26	18 (13%)	8 (31%)	
Bachelor's degree	36	30 (22%)	6 (23%)	
Graduate degree	24	23 (17%)	1 (4%)	
Marital status				0.546
Single	48	39 (29%)	9 (35%)	
Married	75	62 (46%)	13 (50%)	
Previously married	37	33 (25%)	4 (15%)	
Employment				0.651
Full time	43	39 (28%)	4 (15%)	
Part time	16	14 (10%)	2 (8%)	
Retired	24	19 (14%)	5 (19%)	
Homemaker	7	5 (4%)	2 (8%)	
Student	6	6 (4%)	0 (0%)	
STD or LTD	14	11 (8%)	3 (12%)	
SSDI	35	29 (21%)	6 (23%)	
Unemployed	18	14 (10%)	4 (15%)	
Work - pay				0.262
1 to 10 h	7	5 (4%)	2 (8%)	
11 to 20 h	6	5 (4%)	1 (4%)	
21 to 40 h	27	22 (17%)	5 (19%)	
> 40 h	27	26 (20%)	1 (4%)	
None	88	71 (55%)	17 (65%)	
Work - home				0.235
1 to 10 h	96	77 (59%)	19 (73%)	
11 to 20 h	32	26 (20%)	6 (23%)	
21 to 40 h	7	7 (5%)	0 (0%)	
> 40 h	8	7 (5%)	1 (4%)	
None	13	13 (10%)	0 (0%)	
Work - school				0.269
1 to 10 h	9	7 (6%)	2 (8%)	
11 to 20 h	4	2 (2%)	2 (8%)	
21 to 40 h	4	3 (2%)	1 (4%)	
> 40 h	1	1 (1%)	0 (0%)	
None	131	110 (89%)	21 (81%)	
Work - volunteering				0.349
None	117	100 (78%)	17 (65%)	
1 to 10 h	31	23 (18%)	8 (31%)	
11 to 20 h	5	4 (3%)	1 (4%)	
21 to 40 h	2	2 (2%)	0 (0%)	
> 40 h	0	0 (0%)	0 (0%)	
Income				0.122
Comfortable	66	60 (45%)	6 (23%)	
Just enough	55	44 (33%)	11 (42%)	
NOT enough	39	30 (22%)	9 (35%)	
Pain duration				0.655
< 1 years	6	6 (5%)	1 (4%)	
1 to 3 years	15	13 (10%)	2 (8%)	
3 to 5 years	14	11 (8%)	3 (12%)	
5 to 10 years	40	31 (23%)	9 (35%)	
> 10 years	86	75 (55%)	11 (42%)	
Fibromyalgia criteria met				0.252
No	99	89 (62%)	10 (43%)	
Yes	68	55 (38%)	13 (57%)	

*P*-values were calculated using Chi-Squared test.

PRP, pain rehabilitation program; PM&R, physical medicine & rehabilitation; PT, physical therapy; STD, short-term disability; LTD, long-term disability; SSDI, Social Security Disability Insurance.

The difference in referring department between PRP-Yes and PRP-No groups was highly significant (*P* < .001). Specialties with the highest relative representation in PRP-Yes compared to. PRP-No included mental health (21% of PRP-Yes vs. 5% of PRP-No) and rheumatology (17% of PRP-Yes vs. 7% of PRP-No). Specialties with the lowest relative representation in PRP-Yes vs. PRP-No were neurology (10% PRP-Yes vs. 23% PRP-No), interventional pain management (2% PRP-Yes vs. 10% PRP-No), and primary care (13% PRP-Yes vs. 20% PRP-No).

There was a significant difference (*P* = 0.006) in the likelihood to matriculate to PRP between our three PNS medical providers, with one provider making up 71% of the PRP-Yes pool vs. 28% of the PRP-No pool, and another provider representing 2% of the PRP-Yes pool vs. 15% of the PRP-No pool. Similarly, there was a significant difference (*P* = 0.38) in the likelihood to matriculate to PRP between our PT evaluators. The PT provider with highest PRP representation evaluated 78% of PRP-enrollers compared to 67% of PRP-non-enrollers, while the PT provider with the lowest PRP representation evaluated 7% of PRP-enrollers compared to 17% of PRP non-enrollers. There was not a significant difference between PRP-Yes and PRP-No patients depending upon which mental health provider evaluated them.

There were no significant between-group differences in patient demographic variables. The payer mix of patients evaluated was fairly evenly divided between commercial [124 (37%)], Medicaid [98 (29%)], and Medicare [104 (31%)], with an additional 9 (3%) categorized as “other” (mostly self-pay).

While there was no statistical difference in pain (PEG), depression (PHQ), catastrophizing (PCS), physical function (PROMIS-PF) or social function (PROMIS-SF) scores, PRP-enrollers had significantly higher anxiety scores [GAD 9.8 (6.4) vs. 7.0 (6.3), *P* = .018], higher insomnia scores [ISI 17.2 (6.9) vs. 14.1 (7.3), *P* = .045], and higher PTSD scores [2.9 (1.7) vs. 2.0 (2.0), *P* = 0.15] than non-enrollers. While there was not a significant difference between fibromyalgia diagnoses or WPI scores, PRP-enrollers scored significantly higher on the SSS [8.0 (2.4) vs. 6.6 (2.5), *P* = .019].

## Discussion

Our study had several key findings. First, PRP-enrollers were more likely to have had mental health and PT evaluations than non-enrollers. Second, PRP-enrollers had shorter lag times between their initial referral and PNS appointment than non-enrollers. Third, referral source differed significantly between PRP-enrollers and non-enrollers. Fourth, PRP-enrollers differed significantly from non-enrollers in the medical and PT provider seen. Fifth, PRP-enrollers had significantly higher anxiety, PTSD symptoms, somatic symptoms, and insomnia compared to non-enrollers. Finally, PRP enrollment was not significantly impacted by measured demographic characteristics, including race, insurance, or distance from the clinic.

The finding that PRP-enrollers were more likely than non-enrollers to have had mental health, PT, and all three visits is not surprising. Since the PRP includes both psychology and PT services, these initial evaluations are typically required for insurance authorization for patients to participate in PRP. Additionally, our team relies on these evaluations to help determine whether a patient is cognitively and physically appropriate for the rigors of the PRP, with decisions about PRP-enrollment typically being made after a discussion among the three providers. While unsurprising, this finding highlights that failure to receive all three evaluations is a barrier to getting multidisciplinary rehabilitation. Indeed, despite our program being designed to provide all three of these assessments, less than half of our sample (41%) received all three. While we cannot draw definitive conclusions on why this occurred, we suspect that logistics play an important role. Since seeing this data, we have moved to a system in which all three appointments occur on the same day, with a built-in multidisciplinary conference at the end of clinic to determine treatment recommendations (such as whether a patient is appropriate for PRP). We hope that this adjustment will improve both the rate of patients receiving all three assessments and PRP enrollment.

Not only did the number of evaluations contribute to PRP enrollment, but the lag time between initial referral and the PNS medical evaluation was shorter for PRP-enrollers. This is consistent with prior findings on lag time. Shorter lag time has been associated with better pain treatment outcomes (31). Conversely, extensive waiting times have been shown to increase patient burden and decrease patient satisfaction (32, 33). Strategies to mitigate lag times may improve PRP enrollment in the future.

More surprising was the finding that referring department impacted PRP-attendance. First, the diversity of referral sources was unexpected. When the Pain Navigation Service was first developed, the vision was to create a “one-stop-shop” referral destination for primary care. Since we are only tracking those referrals deemed appropriate for PNS by trained nursing triage, it is possible that we are receiving more primary care referrals that are more appropriately routed to other services and therefore not represented in this dataset. For example, primary care may be more likely to send a wide range of referrals that might be more appropriately treated by other specialties, such as neurology or sports medicine, while referrals coming from other specialties may be more targeted to the services offered at PNS. This could also explain why primary care referrals are under-represented in the PRP-attenders. It is less clear why referrals from neurology and interventional pain management are underrepresented in PRP-enrollers. The high representation of mental health referrals in PRP-attendees may suggest that such providers are more successfully able to identify patients who are not only appropriate for such services, but who may also be appropriately motivated to participate in them. Taken together, these findings raise the possibility that improving the PNS marketing and education for primary care providers may lead to more appropriate referrals. Alternatively, an expansion of mental health services into primary care, as can be accomplished through collaborative care models (34, 35), may lead to better patient selection and referral.

Regarding the association between specific medical and PT evaluators - but not mental health evaluators - with PRP enrollment, there are many potential provider-associated factors that could contribute to this (e.g., training background, number of years with the program, level of involvement in the PRP, personality traits, etc.).With such a small number of providers among whom to compare, and without a systematic approach to collect and categorize these different factors, we cannot speculate on which provider characteristics likely contribute to this effect. However, given the robustness of this difference (particularly among medical providers), this result does suggest that individual provider success is a variable worthy of tracking among clinical and research programs, and potentially a topic worthy of targeted research studies.

Considering that measure data was only collected in half of our sample, we should be especially cautious of over-interpreting these findings in this exploratory analysis. With that in mind, it is interesting that pain catastrophizing – the construct most directly addressed by our PRP programming – was not found to be higher in the PRP-enrolling sample. It could be that – since we also have less-intensive options for providing pain psychology – being high in catastrophizing does not necessarily funnel patients toward PRP programming. This finding is consistent with studies conducted in Swedish populations, for whom worse baseline pain outcomes were not associated with enrollment in multimodal pain rehabilitation (36, 37) It is also somewhat surprising that anxiety and PTSD symptoms were significantly higher in our PRP-attendees, as one may have speculated that those symptoms might steer patients away from a 3-week intensive program.

Despite the widespread recognition that socioeconomic and racial disparities contribute to inequities in pain treatment (15, 16), we did not find any differences in PRP-enrollment based on demographic variables, and our programming had a fairly even split between commercial, Medicaid, and Medicare as primary patient insurance. While it is encouraging that our PRP-enrollment does not appear to be excluding particular racial groups – and the proportion of Black patients (15%) is actually higher than the proportion in Indiana by the 2020 census (12%), the proportion of Hispanic patients receiving our services (4%) is considerably lower than the 2020 state census proportion (18%). Factors contributing to this paucity of Hispanic representation in our programming is worthy of further investigation.

### Strengths and limitations

Our utilization of data collected primarily for clinical care is a strength in that it reflects real-world practice and may be more generalizable than data collected in a more controlled setting. However, this approach has several limitations. First, our clinical workflow did not capture information about patients who had been referred to our program but who had been screened out by the nurse prior to entry into the REDCap database. Therefore, we cannot comment on either the number or characteristics of patients screened out at this initial step. Second, we can analyze only those variables that have been collected. There are several key variables that we have not been collecting in a systematic way which are potentially pertinent to this investigation. First would be a variable summarizing whether PRP was recommended after PNS visits, and the reasons why or why not. Knight et al. tracked this information in 200 consecutive patients who received multidisciplinary pain assessment in London, England, and were able to document that 53% were offered treatment, with 35% not offered treatment because they were deemed not ready to change, while 19.5% of patients declined care (38). Adding comprehensive collection of these variables, as well as what other services besides PRP are recommended to patients, is a logical next step in our data collection and analysis. While we cannot make such comparisons with our current dataset, a strength of our approach is that we have data from both the evaluation process and PRP enrollment, so that we can report on whether patients actually made it to the recommended programming. Another key variable we are missing is the out-of-pocket expense that patients might have to pay to enroll in PRP. Anecdotally, this appears to be a major reason why patients decline PRP services and is another variable we hope to capture in the future. Another variable that is not captured is that of patient time availability (i.e., whether patients can commit to a 3-week program). Finally, we did not collect information on patient preferences, perceptions or satisfaction, which have been found to be an important variable that can mitigate program engagement (39, 40). Another limitation to our real-world data approach is that it led to a significant amount of missingness, with nearly half of our sample missing questionnaire data. Despite such a large portion of missingness, questionnaire variables still had >120 respondents each, which provides reasonable power for this exploratory analysis. We presume that clinic logistical factors were the primary driver of this missingness and have used a missing-at-random analysis strategy. However, we cannot rule-out that other factors – i.e., severe clinical depression or cognitive impairment – may have contributed to missingness. While this cannot be reckoned in the present work, we have amended our clinic flow to mitigate this problem in the future by requiring that patients complete the baseline questionnaire before they are able to schedule their PNS visits. We have also combined our two baseline questionnaires into a single questionnaire to reduce redundancy/response burden and simplify analysis for future studies.

## Conclusion

The findings of this study underscore the multifaceted nature of factors influencing enrollment in multidisciplinary pain rehabilitation programs (PRPs). The factors that most strongly predicted PRP enrollment were having undergone both mental health and physical therapy assessments, referring department, and the medical provider seen. While some patient-specific factors, such as anxiety, PTSD symptoms, somatic symptoms, and insomnia, also predicted enrollment, our results suggest that health service variables are also important factors that should be considered in clinical practice and research interested in improving access to PRP care. Given the multiple comparisons made in this study, findings of only marginal significance should be interpreted as exploratory. While the limited geographical extent of the clinic involved in this study may limit generalizability, these results from a conventional US health system complement prior studies from the US VHA system and European systems, with findings overall in harmony with work in those studies.

## Data Availability

The original contributions presented in the study are included in the article/Supplementary Material, further inquiries can be directed to the corresponding author.
