# Supplementary material for: Patient and health service factors associated with enrollment in a multidisciplinary pain rehabilitation program: a retrospective cohort study
Source: Front Pain Res (Lausanne). 2025 Apr 10;6:1455792. doi: 10.3389/fpain.2025.1455792 (PMC12018369; doi:10.3389/fpain.2025.1455792)
Supplement: Supplementary file 1 [file Datasheet1.docx]

STROBE Statement—Checklist of items that should be included in reports of ***cohort studies***

|  | Item No | Recommendation |
| --- | --- | --- |
| **Title and abstract** | 1 | (*a*) Indicate the study’s design with a commonly used term in the title or the abstract  “Retrospective Cohort Study” is indicated in the title and Abstract section. |
|  |  | (*b*) Provide in the abstract an informative and balanced summary of what was done and what was found  Provided in the Abstract section. |
| Introduction | | |
| Background/rationale | 2 | Explain the scientific background and rationale for the investigation being reported  Included in the Introduction section. |
| Objectives | 3 | State specific objectives, including any prespecified hypotheses  Included in the Introduction section. |
| Methods | | |
| Study design | 4 | Present key elements of study design early in the paper  Described in the “Study Design” subsection of the Methods section. |
| Setting | 5 | Describe the setting, locations, and relevant dates, including periods of recruitment, exposure, follow-up, and data collection  Provided in the “Setting” subsection of the Methods section. |
| Participants | 6 | (*a*) Give the eligibility criteria, and the sources and methods of selection of participants. Describe methods of follow-up  Described in the “Participants” subsection of the Methods section. Follow-up was not applicable to this study. |
|  |  | (*b*) For matched studies, give matching criteria and number of exposed and unexposed  Not applicable. |
| Variables | 7 | Clearly define all outcomes, exposures, predictors, potential confounders, and effect modifiers. Give diagnostic criteria, if applicable  This information is included in the “Variable” subsection of the Methods section. |
| Data sources/ measurement | 8* | For each variable of interest, give sources of data and details of methods of assessment (measurement). Describe comparability of assessment methods if there is more than one group  This information is included in the “Data Sources” subsection of the Methods section. |
| Bias | 9 | Describe any efforts to address potential sources of bias  This is described in the “Participants” section of the Methods section. |
| Study size | 10 | Explain how the study size was arrived at  This is described in the “Study Design” subsection of the Methods section. |
| Quantitative variables | 11 | Explain how quantitative variables were handled in the analyses. If applicable, describe which groupings were chosen and why  This information is covered in the “Variables” section of the Methods section. There was no grouping of quantitative variables. |
| Statistical methods | 12 | (*a*) Describe all statistical methods, including those used to control for confounding  This is described in the “Statistical Methods” subsection of the Methods section. |
|  |  | (*b*) Describe any methods used to examine subgroups and interactions  Not applicable. |
|  |  | (*c*) Explain how missing data were addressed  This is described in the “Statistical Methods” subsection of the Methods section. |
|  |  | (*d*) If applicable, explain how loss to follow-up was addressed  Not applicable. |
|  |  | (*e*) Describe any sensitivity analyses  Not applicable. |
| Results | | |
| Participants | 13* | (a) Report numbers of individuals at each stage of study—eg numbers potentially eligible, examined for eligibility, confirmed eligible, included in the study, completing follow-up, and analysed  This is described in the “Participants” subsection of the Methods section. |
|  |  | (b) Give reasons for non-participation at each stage  This is described in the “Participants” subsection of the Methods section. |
|  |  | (c) Consider use of a flow diagram  A flow diagram is provided in Figure 1. |
| Descriptive data | 14* | (a) Give characteristics of study participants (eg demographic, clinical, social) and information on exposures and potential confounders  Where applicable, this information is described in the “Participants” subsection of the Methods section. |
|  |  | (b) Indicate number of participants with missing data for each variable of interest  Number of participants for each variable of interest are provided within Tables 1 & 2. |
|  |  | (c) Summarise follow-up time (eg, average and total amount)  Not applicable. |
| Outcome data | 15* | Report numbers of outcome events or summary measures over time  Not applicable – outcomes were dichotomous and were not measured over time. |
| Main results | 16 | (*a*) Give unadjusted estimates and, if applicable, confounder-adjusted estimates and their precision (eg, 95% confidence interval). Make clear which confounders were adjusted for and why they were included  This information is provided in the “Main results” subsection of the Results section. Since this was a study to identify potential predictors of the PRP enrollment outcome, we did not adjust for confounders. |
|  |  | (*b*) Report category boundaries when continuous variables were categorized  Not applicable. |
|  |  | (*c*) If relevant, consider translating estimates of relative risk into absolute risk for a meaningful time period  Not applicable. |
| Other analyses | 17 | Report other analyses done—eg analyses of subgroups and interactions, and sensitivity analyses  Not applicable. |
| Discussion | | |
| Key results | 18 | Summarise key results with reference to study objectives  This is described in the first paragraph of the Discussion section. |
| Limitations | 19 | Discuss limitations of the study, taking into account sources of potential bias or imprecision. Discuss both direction and magnitude of any potential bias  This is described in the “Strengths and limitations” subsection of the Discussion section. |
| Interpretation | 20 | Give a cautious overall interpretation of results considering objectives, limitations, multiplicity of analyses, results from similar studies, and other relevant evidence  Discussed in the “Conclusion” subsection of the Discussion section. |
| Generalisability | 21 | Discuss the generalisability (external validity) of the study results  Discussed in the “Conclulsion” subsection of the Discussion section. |
| Other information | | |
| Funding | 22 | Give the source of funding and the role of the funders for the present study and, if applicable, for the original study on which the present article is based  Provided in the Funding section that follows the Discussion section. |

*Give information separately for exposed and unexposed groups.

**Note:** An Explanation and Elaboration article discusses each checklist item and gives methodological background and published examples of transparent reporting. The STROBE checklist is best used in conjunction with this article (freely available on the Web sites of PLoS Medicine at http://www.plosmedicine.org/, Annals of Internal Medicine at http://www.annals.org/, and Epidemiology at http://www.epidem.com/). Information on the STROBE Initiative is available at http://www.strobe-statement.org.
