# Supplementary material for: Patient and health service factors associated with enrollment in a multidisciplinary pain rehabilitation program: a retrospective cohort study
Source: Front Pain Res (Lausanne). 2025 Apr 10;6:1455792. doi: 10.3389/fpain.2025.1455792 (PMC12018369; doi:10.3389/fpain.2025.1455792)
Supplement: Supplementary file 2 [file Table1.docx]

| 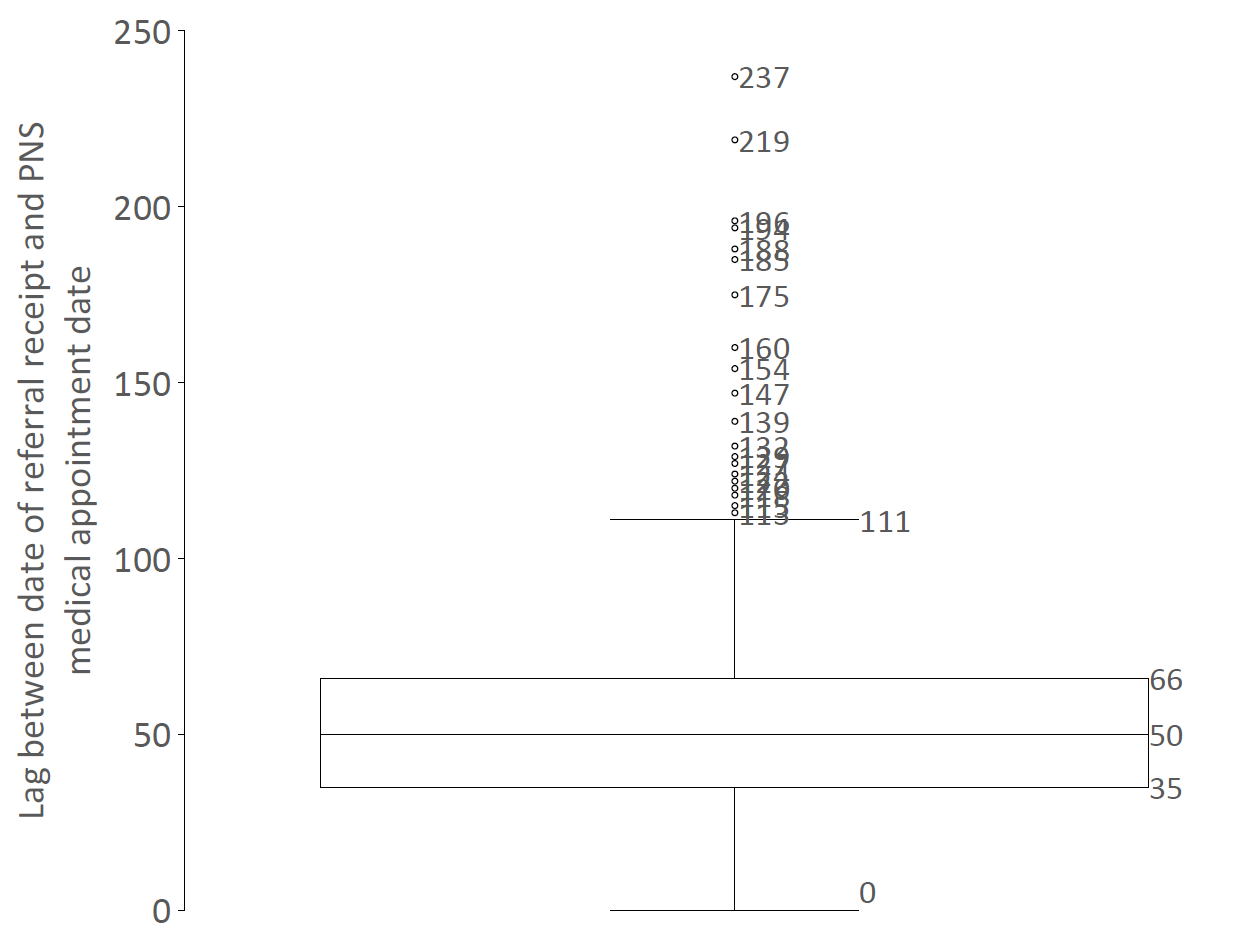 |
| --- |
| **Supplemental Figure**. Box and Whisker Plot of Lag between Referral Receipt Date and Pain Navigation Service (PNS) Medical Visit date for patients who had a PNS medical visit in 2023 (n = 335). Since the minimum to maximum range (excluding outliers) was 0 – 111 days, we chose a 4-month lookback window (i.e. September to December 2022) to encompass the scheduling window for 2023, while excluding those patients that had been scheduled in 2022. |
